# Community-Based Teledermatology for Urgent Suspected Skin Cancer: Health Economic Cost-Comparison and Discrete Event Simulation Study

**DOI:** 10.2196/86402

**Published:** 2026-04-06

**Authors:** Tim C H Hoogenboom, Pablo García Martínez, Piyush Mahapatra, Nurul Ain Nizar

**Affiliations:** 1 Open Medical Ltd, 97-107 Uxbridge Rd, London, W5 5TL, United Kingdom, 44 020 3475 2955; 2 Department of Dermatology, Salford Care Organisation part of the Northern Care Alliance NHS Foundation Trust, Manchester, UK, Manchester, United Kingdom

**Keywords:** teledermatology, urgent suspected cancer, cost-effectiveness, discrete event simulation, patient waiting times, community-based, NHS, National Health Service

## Abstract

**Background:**

The increasing incidence and financial burden of skin cancer place immense pressure on the UK’s National Health Service (NHS). Systemic challenges, including dermatologist shortages and long waiting lists, complicate timely assessment of skin lesions for patients under the urgent suspected cancer pathway. While teledermatology offers an innovative solution compared to traditional face-to-face appointments, standard teledermatology models still face limitations in addressing health care access barriers. Community-based decentralized models may reduce such barriers, but the cost and operational impact of such specific models remain largely underresearched.

**Objective:**

This study evaluated the differences in financial cost to the NHS and patient waiting times at the Northern Care Alliance NHS Foundation Trust by comparing a community-based teledermatology model using Pathpoint eDerma against the Trust’s standard-of-care for patients in the urgent suspected skin cancer pathway.

**Methods:**

This study used an ambidirectional design involving 2 distinct analyses. The cost comparison analysis (CCA) compared costs incurred under the teledermatology model (intervention arm, n=563) against the Trust’s standard care, represented by a synthetic comparator arm (n=4011). The discrete event simulation (DES) modeled the operational impact on patient waiting times over a 1-year period. Data for the intervention arm were collected prospectively from December 2022 to May 2023 for CCA and up to November 2023 for DES, while comparator data were collected retrospectively from September 2021 to December 2022. Publicly available resource costs were incorporated to ensure the robustness of the analyses.

**Results:**

The community-based teledermatology model was associated with significant improvements in both cost to the NHS and patient waiting times. The CCA revealed a mean cost saving of £45 (£1=US $1.24) per referral (95% CI £22-£60; *P*<.001). This cost saving was associated with a 26% reduction in the proportion of patients requiring a full diagnostic biopsy, falling from 48% (1925/4011) in standard care to 22% (124/563) in the teledermatology model as well as time savings in face-to-face clinics and administration. Furthermore, the DES demonstrated that, on average, the teledermatology pathways decreased the time to reach a clinical diagnosis by 9.90 (95% CI 9.64-10.16) days; to communicate a diagnosis to patients by 54.18 (95% CI 50.76-57.61) days; and to reach a histopathological diagnosis by 62.8 (95% CI 59.76-65.83) days compared to standard care.

**Conclusions:**

The implementation of the community-based teledermatology model appears to be a highly effective, cost-efficient strategy associated with shortened patient journeys. The intervention showed a faster initial triage phase, but the study identified the histopathology process as the next major systemic constraint that could deter further pathway efficiency. Achieving timely diagnosis for all patients, including those requiring diagnostic biopsies, will necessitate continued strategic investment in innovative technologies to accelerate this downstream process.

## Introduction

The incidence of skin cancer continues to rise steadily in the United Kingdom. The number of nonmelanoma skin cancer diagnoses surpasses the combined total of the 4 most prevalent nonskin cancers: breast, prostate, lung, and colorectal cancer [1], with malignant melanoma currently listed as the fifth most common cancer [2,3]. The increase in public awareness of skin cancers [4] and the lack of training for general practitioners (GPs) to confidently assess skin lesions [5] have also led to a surge in suspicious skin lesion referrals in the past decade [6]. Skin cancer places a significant financial strain on the National Health Service (NHS), with associated costs in England now projected to reach between £338 million (£1=US $1.24) and £465 million, a significant increase from the previous projection of over £180 million in 2020 [4]. Treatment costs for advanced skin cancer, particularly metastatic melanoma, can be substantial, exceeding £200,000 per case, while early-stage disease has a drastically lower health impact and treatment cost, highlighting the importance of early diagnosis both for the patient and the NHS [7,8].

The rising burden necessitates early detection and management, which, in turn, has placed immense pressure on dermatology services. The NHS is already grappling with significant resource constraints and a national shortage of specialist dermatologists [9]. With almost a quarter of consultant posts unfilled, over 380,000 people are waiting more than 18 weeks for a dermatology appointment [9]. The urgent suspected cancer (USC) pathway aims to provide timely access to care and minimize the risk of cancer progression following initial lesion identification [10]. The aim is achieved by prioritizing patients referred under this pathway to meet the 28-day “Faster Diagnosis Standard” (FDS) [10]; however, national statistics reveal a low conversion rate, indicating that many of these skin lesions are benign [11]. The prioritization of referrals under this pathway has strained resources and subsequently delayed care for patients with other serious, noncancerous conditions like eczema and psoriasis [12].

The systemic challenges have led to the growing adoption of teledermatology as an innovative solution to transform service delivery, although the traditional model of lesion assessment through face-to-face (F2F) clinic appointments remains common [10,13]. Teledermatology is most frequently delivered through a store-and-forward (SAF) model, which involves capturing and transmitting skin lesion images and clinical data on digital platforms for remote assessment by dermatologists [14]. A body of evidence has demonstrated the general effectiveness of this SAF model in addressing workforce constraints, reducing waiting times, and improving patient access [15,16].

However, there are operational limitations reported with the conventional SAF model, specifically within the image capture process [10,13,14,17]. Models relying on image capture performed at the secondary care provider level maintain high image quality but limit efficiency gains by requiring patients to travel to a centralized hospital facility [10,13,14,17]. Conversely, referrer (GP) image capture models often suffer from lower image quality, reduced standardization, and limited uptake due to existing constraints on GP capacity [10,13,14,17]. Consequently, the effectiveness of the traditional SAF model is still limited by existing challenges in health care access, including geographical distance and avoidant behaviors that discourage patients from visiting centralized secondary care facilities [10,13,14,17]. Single-site SAF models in particular are also limited in their ability to support regional strategic plans that seek to pool resources and support smaller sites.

To mitigate the challenges inherent in conventional teledermatology, we implemented a community-based teledermatology model, using the Pathpoint eDerma digital platform (Open Medical Ltd). This model was specifically designed to ensure high-quality image capture delivered by a dedicated health care assistant while providing timely and accessible care closer to patients’ homes. This approach aligns directly with the NHS’s strategic vision to shift “from hospital to community,” thereby improving access, quality, standardization, and uptake of the teledermatology service [18]. The platform’s data architecture and integration capabilities are also future-proofed to support delivery of more comprehensive regional care models [19].

Currently, the literature lacks a robust, real-world evaluation of a community-based model within the urgent suspected skin cancer pathway in the NHS. This study sought to address this gap by providing a comprehensive analysis of the cost implications and operational efficiency of the community-based teledermatology model from a health care system standpoint. In this study, we compare a community-based teledermatology model using Pathpoint eDerma against the trust’s standard of care for patients in the urgent suspected skin cancer pathway to evaluate the associated cost implications for the Northern Care Alliance NHS Foundation Trust (NCAFT) and patients’ waiting times.

## Methods

### Study Setting

The study was conducted within the dermatology department at NCAFT. This trust is one of the largest NHS providers in the country, which delivers health care to over 1 million people across Salford, Oldham, Rochdale, and Bury [20]. As part of a wider teledermatology project launched in December 2022, NCAFT established its first community-based teledermatology service. This model used a community diagnostic center (CDC) in Bury, allowing patients to have their skin lesions photographed closer to home rather than at the traditional location, Salford Royal Hospital. The study population included patients referred by their GPs to the urgent suspected skin cancer pathway (formerly known as the 2-week wait [2WW]), who presented with 2 or fewer suspicious lesions.

### Study Design

This study used a comparative, ambidirectional design to evaluate a teledermatology model against local standard care. It consisted of 2 distinct analyses: a cost comparison analysis (CCA) to evaluate the budgetary implications for the NHS trust and a discrete event simulation (DES) to model the differences in patient waiting times. The study is considered ambidirectional as data for the intervention arm were collected prospectively, while data for the comparator arm were collected retrospectively from historical records. Different time horizons were used in the analyses and are detailed in the data collection section.

### Intervention and Comparator Arms

Each arm of the study comprised several distinct pathways representing the patient journey from referral to diagnosis. Under the intervention arm, a patient’s journey began with an image capture appointment at the CDC. There, a trained health care assistant captured high-quality macroscopic and dermatoscopic images of the lesion(s) and uploaded them, along with referral notes and a digitally completed patient questionnaire, to the eDerma platform. A consultant dermatologist then remotely reviewed this information to determine the clinical outcome. This process defined 5 distinct intervention pathways (A-E).

**Figure 1.**
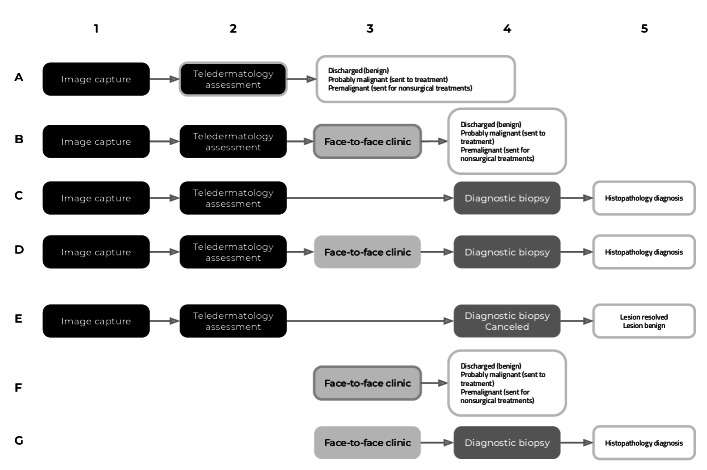
Potential pathways under the intervention and comparator arms.

In contrast, the comparator arm reflected the local standard-of-care pathways (F and G), where patients attended a F2F clinical appointment with a consultant dermatologist for an in-person dermoscopic examination. Following this consultation, the patient was either discharged (pathway F) or scheduled for a diagnostic biopsy (pathway G). The outcome of a canceled diagnostic biopsy (pathway E) is also a potential event for patients following the standard-of-care F2F pathway, though less frequent and was not observed in the dataset used for this study (Figure 1).

### Data Collection

For the CCA, prospective data for the teledermatology model’s intervention arm were sourced from the Pathpoint eDerma platform, spanning from December 2022 to May 2023. Conversely, retrospective data for the standard care comparator arm were collected from historical NCAFT records from September 2021 to December 2022 and supplemented with public NHS protocols. This process created a synthetic control group for the comparator arm, enabling a direct cost comparison between the 2 pathways. Due to the historical nature of the records, this cohort was reconstructed using a triangulated audit approach. A manual review of 70 F2F clinical cases was conducted to determine granular pathway distributions (F and G), which were then calibrated against a larger trust-level dataset of over 5000 urgent referrals to establish representative waiting times. The comparator arm’s period was also chosen to ensure it reflected a post-lockdown health care environment. National NHS data indicated that by late 2021, USC referrals for skin lesions had returned to, or exceeded, prepandemic volumes [21]. Consequently, the analysis assumes that patient reluctance to seek treatment was not a significant factor in the diagnostic timelines observed during the comparator period. Additionally, we included resource utilization data, such as staff labor and procedure costs (eg, diagnostic biopsies), which were adjusted to 2023 values for inflation using the Personal Social Services Research Unit manual [22] and the NHS National Cost Collection schedule [23].

The same retrospective data were then repurposed for the DES to inform the operational parameters of the “as-is” model, effectively defining the simulation’s structure and serving as the baseline for comparison. To increase the real-world accuracy of the simulation, we integrated additional data to define specific parameters. These included patient pathway proportions and communication methods from the eDerma platform, patient inflow rates from NHS England’s Cancer Waiting Times Statistics [21], and staff numbers estimated from NHS Hospital & Community Health Service monthly workforce statistics [24]. The simulation of the intervention arm was further strengthened by incorporating an additional 6 months of prospective data, extending its data collection period up to November 2023.

### Statistical Analyses

To ensure the study’s independence and meet the requirements of the teledermatology project’s funders, the CCA and DES were conducted by an external health technology consultancy, Health Tech Enterprise. Although the analyses were performed independently, the authors of this study provided the necessary datasets and contextual information. They also reviewed the methodology to ensure it accurately reflected the specific teledermatology project under evaluation.

The health economic CCA compared and quantified the budgetary impact of the teledermatology model against standard care from the perspective of the NHS. The analysis focused on direct medical costs incurred from the point of referral to the point of diagnosis, including health care staff labor (Bands 3, 5, 9, and Consultant levels), diagnostic biopsy procedures, and technology platform licensing. A health economic model was constructed in Microsoft Excel (Microsoft 365, v2310; Microsoft Corp) to determine the average cost per referral for both the intervention and comparator arms. This was followed by an incremental analysis to account for the SEs associated with health care staff involvement.

To test whether the difference in mean costs was statistically significant, an unpaired *t* test was performed. Further, we conducted a probability sensitivity analysis with 1000 model iterations and a deterministic sensitivity analysis to account for uncertainty in input parameters and evaluate how changes in key parameters, such as the number of biopsies, affected the results.

Moreover, we performed a DES to evaluate the impact on patient waiting times. The skin cancer screening pathways were modeled as an agent-based DES using SIMUL8 software (Professional Edition) over a 1-year time horizon. The model’s capacity was based on the availability of health care staff and accounted for the seasonality of patient inflow. The simulation was run for five 1-year instances, with each patient “agent” entering the model upon referral and exiting once a diagnosis was communicated.

The simulation measured 3 key end points, representing different waiting periods: the average time from referral to clinical diagnosis, the average time from referral to histopathological diagnosis (for patients requiring a diagnostic biopsy), and the average time to final diagnosis communication.

### Model Assumptions

The CCA was built upon several assumptions regarding patient characteristics and resource utilization to enable a direct cost comparison between the intervention and comparator pathways. It was assumed that patient characteristics were the same in both the intervention and comparator arms. For the standard-of-care (comparator) arm, all consultations were modeled as F2F. Specific durations were assigned to clinical activities; the initial F2F consultation with a consultant dermatologist was set at 20 minutes, while a follow-up clinic appointment after the eDerma assessment was set at 15 minutes. The resource cost model assumed that a photography appointment, conducted by an NHS Band 3 health care assistant at a local GP practice, took 20 minutes, and the remote eDerma assessment by a consultant dermatologist took approximately 4.97 minutes. Furthermore, it was assumed that any dermoscopy examination performed in the comparator arm was included within the F2F consultation time, eliminating the need for an additional photography assessment.

Similarly, the DES relied on several assumptions to model patient flow and system capacity under controlled conditions. The simulation modeled high-risk patients referred via the 2WW pathway with 2 or fewer lesions, reflecting current clinical practice and ensuring a realistic assessment of the current adoption of eDerma technology in the health care setting. This approach provides a baseline understanding of the technology’s impact under current conditions and practices.

The simulation also assumed 100% patient retention (no dropouts) and no patient “no shows” for F2F appointments. The model also excluded patient mortality. Operational capacity was modeled based solely on health care staff availability, assuming fixed staffing levels throughout the simulation and excluding equipment or facility constraints. Furthermore, labor was scheduled with staff available on weekdays (8:30 AM to 6:30 PM) and 70% of staff available for reduced weekend shifts, with the exception of GP. Procedures were allocated based on a simplified first-come, first-served basis and patient risk level, disregarding individual patient availability.

### Ethical Consideration

The study used anonymized standard care data. The project was reviewed by the NCAFT Research and Innovation Department (22HIP55) and was determined not to require further ethical review. Specifically, the project was registered as a Health Improvement Project (22HIP55) and was determined by the Research and Innovation office not to require NHS Health Research Authority review. This determination was made in accordance with national regulations, including the Data Protection Act [25], the General Data Protection Regulation [26], and applicable Health Research Authority [27] guidance. As this was a retrospective study using anonymized routinely collected data, the requirement for formal informed consent was not required, as confirmed by the NCA Research and Innovation department. For the Patient-Reported Experience Measure surveys, patients were informed of the evaluation’s purpose, and participation was entirely voluntary. No compensation or financial incentives were provided to the patients participating in the surveys. To ensure patient privacy, all data were deidentified before being used for analysis under the formal Data Sharing and Processing Agreements. Furthermore, all researchers were issued formal Letters of Access mandating strict adherence to the trust’s information governance and confidentiality policies, and all methods in this study adhered to the ethical principles outlined in the Declaration of Helsinki.

## Results

### Description of Dataset

This study’s dataset included 563 urgent skin lesion referrals (so-called “2WW” referrals) managed via the intervention arm. A synthetic comparator arm of 4011 referrals was generated based on referral-to-diagnosis times from 2 years of urgent skin cancer referrals (>5000 cases), supplemented with more granular information regarding the methods of diagnosis (F and G in Figure 1) that were available from 70 cases. The same data distributions were subsequently used to conduct the DES.

Within the intervention arm cohort, 324 out of 563 (57.5%) were female participants, and this cohort had a mean age of 61.5 (SD 17.6) years at the time of referral (Table 1), consistent with the higher risk of skin cancer in older populations resulting from accumulated, lifelong UV exposure [1,4,10]. While granular demographic data for the synthetic comparator arm were unavailable, clinical comparability between the 2 arms was maintained by ensuring that both cohorts consisted of adult patients referred via the USC pathway, presenting with 2 or fewer suspicious lesions at the trust. Furthermore, a parallel health inequality assessment, conducted independently of the CCA and DES but within the same trust and clinical pathway, confirmed that the local patient population remains sociodemographically consistent. The NCA cohort typically reflects a more socioeconomically advantaged profile, with a median Index of Multiple Deprivation decile of 6 (IQR 3-8) and a mode of 8.

**Table 1.** Sociodemographic and clinical characteristics of the intervention arm (N=563).

Characteristic	Value
Sex, n (%)	
Female	324 (57.5)
Male	239 (42.5)
Age (y), mean (SD)	61.5 (17.6)
Referral pathway	Urgent suspected cancer with 2 or fewer suspicious lesions

### Cost Comparison Analysis

The analysis demonstrates substantial cost savings for the NCAFT through the implementation of the community-based teledermatology model compared to standard care (Table 2). The probability sensitivity analysis showed a mean savings of £45 (95% CI £22-£60) per referral with the eDerma model. This translates to a potential overall savings of £25,251 (95% CI £12,462-£34,002) for the NCAFT from December 2022 to May 2023. An unpaired *t* test confirmed that the difference in mean costs between the 2 arms was statistically significant (*P*<.001). Based on 1000 iterations, a cost reduction per referral was observed in 94% of cases.

A subanalysis further revealed the mechanism behind these savings by assessing the distribution of referrals and their associated unit costs (Table 3). The highest costs were incurred in pathways involving a full diagnostic biopsy, specifically pathways C, D, and G. The community-based eDerma model led to an 18% reduction in referrals needing a biopsy, as the percentage of patients in biopsy-reliant pathways fell from 49% (1965/4011) in the comparator arm (pathway G) to 31% (175/563) in the intervention arm (pathways C and D combined). A 1-way deterministic sensitivity analysis supported this finding by showing that if the eDerma system could offset the need for full biopsies, it could lead to a mean cost savings of up to £135 per referral.

**Table 2.** Potential cost savings via the eDerma intervention arm.

Economic parameter	Cost savings^a^ (comparator arm–eDerma)	95% CI
Potential cost savings per referral	£45	£22-£60
Potential overall cost savings	£25,251	£12,462-£34,002

a £1=US $1.24.

**Table 3.** Referral percentage and unit costs in each pathway.

Arms and associated pathways	Referral percentage (%)	Unit cost (£)^a^
Intervention arm (eDerma)		
A	44	55
B	21	219
C	17	371
D	14	542
E	3	137
Comparator arm (standard-of-care)		
F	51	163
G	49	364

a £1=US $1.24.

### Discrete Event Simulation

The DES used a slightly different pathway distribution due to the additional 6 months data for the intervention arm. The simulation demonstrated a similar and even more pronounced effect, with the biopsy rate falling from 48% (1925/4011) (pathway G) in standard care to 22% (pathway C and D) in the teledermatology model (Table 4).

**Table 4.** Pathway distribution used in the discrete event simulation.

Arms and associated pathways	Referral percentage (%)
Intervention arm	
A	53
B	24
C	5
D	17
E	1
Comparator arm	
F	52
G	48

#### Referral to Diagnosis Communication

To determine the overall average waiting times for the eDerma and standard-of-care arms, a weighted average was calculated by combining the average time for each pathway (A-E in the intervention arm vs F and G in the comparator arm) with the proportion of patients using each communication method. Finally, an incremental analysis was performed to compare the average waiting times of the 2 arms.

The methods of diagnosis communication included letters, emails via the eDerma platform, telephone calls, and F2F appointments. Complete results on the average time for each communication method and the weighted average of each pathway are detailed in Multimedia Appendix 1.

The analysis of weighted average times per pathway reveals that pathway A in the intervention arm has the shortest average time from referral to diagnosis communication, at 8 days. In contrast, the pathways involving diagnostic biopsies, pathways C and D from the intervention arm and pathway G from the comparator arm, show substantially longer weighted average times of 52.4 and 131.1 days, respectively.

The overall weighted average time for the intervention arm was 18.97 (SE 0.92) days. Conversely, the standard care arm had a weighted average time of 73.16 (SE 1.48) days, illustrating a more extended duration (Table 5).

**Table 5.** Comparison of the overall weighted average time from referral to diagnosis communication.

Referral to diagnosis (d)	Intervention arm	Comparator arm
Weighted average time	18.97	73.16
Weighted standard error	0.92	1.48
Maximum time	109.5	252.3

#### Referral to Clinical Diagnosis

The DES analysis demonstrated that the eDerma arm reached clinical diagnosis faster, with a mean of 7.38 (95% CI 7.24-7.52) days from the initial referral. In the comparator arm, the mean time to clinical diagnosis was longer, at 17.29 (95% CI 17.07-17.50) days.

#### Referral to Histopathological Diagnosis

For patients undergoing a biopsy, the histopathological diagnosis occurred, on average, within 66.42 (95% CI 65.33-67.50) days in the eDerma arm (Table 6). This was significantly shorter than the comparator arm, where the average waiting period for a histopathological diagnosis was 129.21 (95% CI 126.38-132.05) days (Table 6).

A subsequent incremental analysis highlighted the impact of the community-based teledermatology model in reducing patients’ waiting times. On average, the teledermatology pathways were associated with a decrease in the time required to establish and communicate a skin cancer diagnosis to a patient by 54.18 (95% CI 50.76 to 57.61) days. Furthermore, the average waiting time for a clinical diagnosis and histopathological diagnosis was reduced by 9.90 (95% CI 9.64-10.16) days and 62.80 (95% CI 59.76-65.83) days, respectively. These findings are summarized in Table 7.

**Table 6.** Comparison of referral to clinical diagnosis and referral to histopathological diagnosis results.

Parameter	Intervention arm (teledermatology model)	Comparator arm (standard care)
Referral to clinical diagnosis (d)		
Mean (SE)	7.38 (0.07)	17.29 (0.11)
95% CI	7.24-7.52	17.07-17.50
Max	31.67	50.28
Referral to histopathological diagnosis (d)		
Mean (SE)	66.42 (0.55)	129.21 (1.45)
95% CI	65.33-67.50	126.38-132.05
Max	109.41	248.37

**Table 7.** Summary of discrete event simulation analysis.

Incremental analysis Δ (waiting time in current care–waiting time in eDerma care)	Mean (95% CI)
Average time to diagnosis communication (d)	54.18 (50.76-57.61)
Average time to clinical diagnosis (d)	9.90 (9.64-10.16)
Average time to histopathological diagnosis (d)	62.80 (59.76-65.83)

## Discussion

### Principal Findings

The study demonstrated that the community-based teledermatology model, facilitated by Pathpoint eDerma, was associated with significant cost savings of £45 per referral and a 54.18-day reduction in diagnosis communication. This efficiency is driven by the operational shift from a centralized secondary care workflow to a decentralized, community-level approach. By moving imaging to local CDCs, the model effectively diverted referrals away from hospital-based bottlenecks and addressed the “upstream” consultant-capacity shortages, allowing dermatologists to triage cases remotely.

Another driver of these efficiencies was the 26% reduction in the proportion of patients requiring biopsy-reliant pathways, falling from 48% (1925/4011) in standard care to 22% (124/563) in the teledermatology model. The analysis demonstrates that diagnostic biopsies are the most expensive and resource-intensive stage of the urgent skin cancer journey. By facilitating definitive clinical triage through high-quality imaging, the teledermatology model minimizes unnecessary surgical interventions, potentially leading to cost savings of up to £135 per referral when biopsies are offset.

The model successfully mitigates the initial bottleneck associated with consultant capacity, effectively managing high referral volumes. Our DES highlighted that this upstream optimization has, in turn, revealed a downstream constraint within the histopathology service. The massive disparity in waiting times between pathways requiring a biopsy and those that do not highlights a critical resource limitation at the histopathology level. This constraint now represents the primary rate-limiting step impacting referral-to-diagnosis time. Therefore, to realize further gains in operational efficiency and to ensure that the 28-day FDS is consistently achieved, future service improvement initiatives should be directed toward optimizing the capacity and workflow of the histopathology reporting pathway.

Operational solutions, such as the outsourcing of histopathology services, are currently performed in NCAFT following the completion of this study to increase reporting capacity. Looking forward, this constraint could also potentially be resolved through the adoption of technologies like digital and computational pathology [28,29]. Digital pathology (eg, whole slide imaging) can enable remote review by pathologists [28]. Alternatively, computational pathology, including artificial intelligence, could accelerate diagnostic throughput by providing intelligent triage to prioritize high-risk cases and automated quantification to speed up time-intensive tasks like measuring tumor margins [29].

### Policy Implications

The success of this model has significant implications for NHS health system planning. The findings support the NHS England Teledermatology Roadmap [10] by demonstrating that decentralized models can successfully achieve national targets that remain elusive under traditional F2F care. For these models to be scalable, future policies must prioritize high standards of digital interoperability where platforms can securely span the entire referral journey, enabling swift information sharing between GP, patients, and secondary care providers. Furthermore, the transition “from hospital to community” aligns with the NHS 10-year vision [18], suggesting that future capital investment should be directed toward community imaging infrastructure to sustain these efficiency gains.

### Limitations and Recommendations

As an ambidirectional study, the use of nonoverlapping time horizons between the study arms introduces a potential risk of temporal confounding. Observed improvements in wait times and biopsy rates may have been partially influenced by systemic shifts in NHS dermatology protocols following the October 2022 guidelines. Nonetheless, it is essential to distinguish between the national mandate provided by these guidelines and the operational execution enabled by the intervention. Although the guidelines formalize the 28-day FDS, national dermatology waiting lists have remained at historic highs due to chronic consultant shortages [9]. Furthermore, national data indicate that by late 2021, USC referrals had already reached record-high volumes [21], suggesting that the comparator period (September 2021 to December 2022) represents a stable baseline of high systemic operational pressure. The 54.18-day reduction in diagnosis communication suggests an operational impact that far exceeds typical year-on-year service fluctuations, indicating that the digital model addressed these persistent pressures more effectively than the traditional F2F standard of care.

Another methodological limitation was the exclusion of false-negative rates and their associated long-term treatment costs from the economic model. While a longitudinal follow-up for this specific cohort was not feasible, SAF teledermatology possessed an established safety profile in clinical literature, demonstrating diagnostic accuracy and sensitivity comparable to traditional F2F triage [10,14,15,30]. To ensure a transparent and unbiased economic evaluation, these potential downstream costs were excluded consistently across both study arms. Consequently, the reported £45 mean cost saving represents a conservative estimate of the direct, front-end budgetary impact on the trust.

The DES relied on approximations for parameters such as histopathological processing times and professional review durations, which may not perfectly mirror real-world variability. Additionally, the simulation did not account for missed appointments or fluctuating staff availability, and the single-site nature of the study may limit the immediate generalizability of these findings to other regions. To address these constraints, future research could use multisite longitudinal designs, such as randomized stepped-wedge trials, to assess the long-term sustainability of these cost savings. Given that various teledermatology models are already deployed across the United Kingdom, a collaborative effort to prospectively collect standardized data for comparative health economic evaluations may represent the most pragmatic next step.

Furthermore, the use of a F2F clinic as the comparator, as opposed to a direct comparison with a traditional single-site SAF teledermatology service, was a deliberate choice to evaluate the model against current local standards. It is crucial to note that the community-based model offers inherent strategic advantages not captured by wait-time and financial metrics alone, such as improved patient access [12], reduced patient costs [31], and its unique suitability for deploying region-wide collaborative care networks that single-site models may struggle to support.

From a clinical perspective, this study focused on patients in the USC pathway with 2 or fewer suspicious lesions, a cohort primarily consisting of benign cases or early-stage malignancies. While this pathway is key for early detection, individuals with late-stage skin cancer are less likely to access care through this specific route. Therefore, further research is required to understand the journeys of patients with advanced disease to identify unique barriers and develop targeted interventions for timely access.

Ultimately, the community-based teledermatology model was developed to resolve NHS dermatology capacity constraints and to serve as a robust mitigation strategy for traditional access barriers. While the primary focus of this study was on the budgetary and operational implications, findings from a parallel Health Inequality Assessment at the same trust confirmed that diagnostic timelines remained equitable across all age and deprivation groups within this model. This suggests that decentralizing care to local CDCs mitigates the travel and mobility burdens that traditionally hinder older populations at higher risk of skin cancer [1,4,10] and those from more deprived socioeconomic backgrounds [31].

### Conclusion

Our results suggest that a community-based teledermatology model, using the Pathpoint eDerma platform, can provide an efficient and cost-effective pathway for urgent suspected skin cancer referrals, benefiting both the health care system and patients. By being associated with a 26% reduction in biopsy-reliant pathways and an observed 54.18-day reduction in communication times, the model offers a viable strategy to address critical resource constraints within the NHS. Future practical interventions should focus on expanding community-based imaging infrastructure to reduce travel burdens for high-risk older populations, while exploring the potential of digital pathology and other operational solutions to resolve the remaining downstream bottlenecks identified in this analysis.

## Supplementary material

10.2196/86402 Multimedia Appendix 1 Average time from referral to diagnosis per communication methods and weighted average time per pathway.

## Abbreviations

CCA: cost comparison analysis
CDC: community diagnostic center
DES: discrete event simulation
F2F: face-to-face
FDS: Faster Diagnosis Standard
GP: general practitioner
NCAFT: Northern Care Alliance NHS Foundation Trust
NHS: National Health Service
SAF: store-and-forward
USC: urgent suspected cancer

## References

[1] Non-melanoma skin cancer statistics. Cancer Research UK.

[2] Melanoma skin cancer statistics. Cancer Research UK.

[3] Chen K, Liu X (2025). Global burden of skin cancer and its subtypes: a comprehensive analysis from 1990 to 2021 with projections to 2040. Front Public Health.

[4] Skin cancer campaign. Cancer Research UK.

[5] Shivakumar H, Chanda UL, Onwuchekwa O (2025). Evaluating the diagnostic accuracy and challenges of the two-week wait referral pathway for skin cancers in primary care. Cureus.

[6] Early cancer diagnosis data hub. Early Diagnosis Hub.

[7] Goon PKC, Greenberg DC, Igali L, Levell NJ (2017). Predicted cases of U.K. skin squamous cell carcinoma and basal cell carcinoma in 2020 and 2025: horizon planning for National Health Service dermatology and dermatopathology. Br J Dermatol.

[8] Cutting VAT from sunscreen could save the NHS over £128 million per year in cancer treatment costs. Melanoma Focus.

[9] (2021). Dermatology overview. https://www.gettingitrightfirsttime.co.uk/wp-content/uploads/2021/11/Dermatology-overview.pdf.

[10] A teledermatology roadmap: implementing safe and effective teledermatology triage pathways and processes. National Health Service England.

[11] Diagnostic waiting times and activity. National Health Service England.

[12] (2022). Referral optimisation for people with skin conditions. https://www.england.nhs.uk/wp-content/uploads/2022/09/B1149-referral-optimisation-for-people-with-skin-conditions.pdf.

[13] (2022). Quality standards for teledermatology. https://cdn.bad.org.uk/uploads/2022/02/29200021/Teledermatology-Quality-Standards.pdf.

[14] Jiang SW, Flynn MS, Kwock JT, Nicholas MW (2022). Store-and-forward images in teledermatology: narrative literature review. JMIR Dermatol.

[15] López-Liria R, Valverde-Martínez MÁ, López-Villegas A (2022). Teledermatology versus face-to-face dermatology: an analysis of cost-effectiveness from eight studies from Europe and the United States. Int J Environ Res Public Health.

[16] McKoy K, Halpern S, Mutyambizi K (2021). International teledermatology review. Curr Dermatol Rep.

[17] (2024). Service guidance and standards for the use of teledermatology. https://cdn.bad.org.uk/uploads/2024/12/16150109/Service-Guidance-and-Standards-for-Teledermatology-2024.pdf.

[18] (2019). The NHS long term plan. https://webarchive.nationalarchives.gov.uk/ukgwa/20230418155402/https:/www.longtermplan.nhs.uk/publication/nhs-long-term-plan/.

[19] New digital platform set to transform urgent suspected skin cancer referrals in Lancashire and South Cumbria. Lancashire and South Cumbria Cancer Alliance.

[20] Northern Care Alliance NHS Foundation Trust.

[21] 2022‑23 monthly provider cancer waiting times statistics. National Health Service England.

[22] Jones KC, Weatherly H, Birch S (2022). Unit costs of health and social care 2022 manual. https://kar.kent.ac.uk/100519/1/Unit%20Costs%20of%20health%20and%20Social%20Care%202022%20%28amended%2013%20July%202023%29.pdf.

[23] National cost collection for the NHS. National Health Service England.

[24] (2023). NHS workforce statistics - July 2023. National Health Service England.

[25] Data Protection Act 2018. legislation.gov.uk.

[26] European Parliament and Council of the European Union Regulation (EU) 2016/679 of the European Parliament and of the Council of 27 April 2016 on the protection of natural persons with regard to the processing of personal data and on the free movement of such data (General Data Protection Regulation). http://data.europa.eu/eli/reg/2016/679/oj.

[27] Is my study research?. Health Research Authority.

[28] Zarella MD, McClintock DS, Batra H (2023). Artificial intelligence and digital pathology: clinical promise and deployment considerations. J Med Imaging (Bellingham).

[29] Kim I, Kang K, Song Y, Kim TJ (2022). Application of artificial intelligence in pathology: trends and challenges. Diagnostics (Basel).

[30] Chuchu N, Dinnes J, Takwoingi Y (2018). Teledermatology for diagnosing skin cancer in adults. Cochrane Database Syst Rev.

[31] Nizar NA, Farooki R, Mahapatra P, Halpern S, Hoogenboom TCH (2024). Patient cost analysis of a community-based teledermatology service versus conventional outpatient appointments in East Kent: a retrospective study through a societal lens to reduce health inequalities. BMC Health Serv Res.

