# Supplementary material for: Community-Based Teledermatology for Urgent Suspected Skin Cancer: Health Economic Cost-Comparison and Discrete Event Simulation Study
Source: JMIR Dermatol. 2026 Apr 6;9:e86402. doi: 10.2196/86402 (PMC13053001; doi:10.2196/86402)
Supplement: Multimedia Appendix 1 [file derma-v9-e86402-s001.pdf]

Multimedia Appendix 1

Table 1: Average time from referral to diagnosis per communication methods used in each pathway.

Pathway	Communication method	Average time (days)	Maximum time (days)	Lower 95% CI	Upper 95% CI	Standard error (SE)
A	Letter	7.98	32.24	7.76	8.25	0.13
A	eDerma	8.02	32.22	7.73	8.35	0.16
A	Phone call	7.99	32.26	7.56	8.50	0.24
B	Face-to-face appointment	12.89	49.29	10.51	15.64	1.31
B	Letter	11.72	47.52	10.07	13.41	0.85
B	Phone call	12.99	46.87	10.21	16.28	1.55
C/D	Face-to-face appointment	52.38	109.48	44.35	59.86	3.95
C/D	Letter	51.91	108.96	44.38	58.53	3.61
C/D	Phone call	53.33	108.39	45.67	60.44	3.77
F	Face-to-face appointment	19.65	53.39	18.71	20.78	0.53
F	Letter	19.57	53.06	18.74	20.60	0.48
F	Phone call	19.47	51.38	18.63	20.41	0.46
F	Email	20.06	51.32	18.12	22.59	1.14
G	Face-to-face appointment	131.34	252.30	124.37	136.17	3.01
G	Letter	129.79	252.01	118.37	137.45	4.87
G	Email	132.81	252.19	119.37	142.27	5.84
G	Phone call	125.02	251.54	111.26	140.90	7.56

Table 2: Weighted average time from referral to diagnosis communication per pathways.

Pathway	Weighted average time (days)	Weighted Standard Error (WSE)	Maximum time (days)
A	8.0	0.1	32.3
B	12.9	1.3	49.3

C/D	52.4	3.9	109.5
E	12.9	1.8	53.6
F	19.7	0.5	53.4
G	131.1	3.0	252.3
